# Understanding the sources of performance in deep drug response models reveals insights and improvements

**DOI:** 10.1093/bioinformatics/btaf255

**Published:** 2025-07-15

**Authors:** Nikhil Branson, Pedro R Cutillas, Conrad Bessant

**Affiliations:** School of Biological and Behavioural Sciences, Queen Mary University of London, London E1 4NS, United Kingdom; Digital Environment Research Institute, Queen Mary University of London, London E1 1HH, United Kingdom; Centre for Genomics and Computational Biology, Barts Cancer Institute, Queen Mary University of London, London EC1M 6BQ, United Kingdom; School of Biological and Behavioural Sciences, Queen Mary University of London, London E1 4NS, United Kingdom; Digital Environment Research Institute, Queen Mary University of London, London E1 1HH, United Kingdom

## Abstract

**Motivation:**

Anti-cancer drug response prediction (DRP) using cancer cell lines (CLs) is crucial in stratified medicine and drug discovery. Recently, new deep learning models for DRP have improved performance over their predecessors. However, different models use different input data types and architectures making it hard to find the source of these improvements. Here we consider published DRP models that report state-of-the-art performance predicting continuous response values. These models take chemical structures of drugs and omics profiles of CLs as input.

**Results:**

By experimenting with these models and comparing with our simple baselines, we show that no performance comes from drug features, instead, performance is due to the transcriptomics CL profiles. Furthermore, we show that, depending on the testing type, much of the current reported performance is a property of the training target values. We address these limitations by creating BinaryET and BinaryCB that predict binary drug response values, guided by the hypothesis that this reduces the noise in the drug efficacy data. Thus, better aligning them with biochemistry that can be learnt from the input data. BinaryCB leverages a chemical foundation model, while BinaryET is trained from scratch using a transformer-type architecture. We show that these models learn useful chemical drug features, which is the first time this has been demonstrated for multiple testing types to our knowledge. We further show binarizing the drug response values causes the models to learn useful chemical drug features. We also show that BinaryET improves performance over BinaryCB, and the published models that report state-of-the-art performance.

**Availability and implementation:**

Code is available from https://github.com/Nik-BB/Understanding_DRP_models.

## 1 Introduction

Anti-cancer drug response prediction (DRP) has three main aims that, if delivered, would each improve patient outcomes or decrease treatment costs. These aims are to: (i) repurpose existing drugs, (ii) tailor more effective treatments to specific groups or individuals and (iii) help discover novel drugs. Cancer cell lines are at the heart of most DRP studies because they give a proxy for patient data while offering an abundance of publicly available drug screening data (Sharifi-Noghabi *et al.* 2021b). Several large-scale public databases provide drug response measurements and omics cell line profiles. These include the Genomics of Drug Sensitivity in Cancer (GDSC) (Yang *et al.* 2013), the cancer cell line encyclopedia (CCLE) and the Cancer Therapeutic Response Portal (CTRP) (Seashore-Ludlow *et al.* 2015). See Baptista *et al.* (2021) and Sharifi-Noghabi *et al.* (2021b) for in-depth comparisons of the different databases. These databases typically contain hundreds of thousands of drug response measurements.

**Figure 1.**
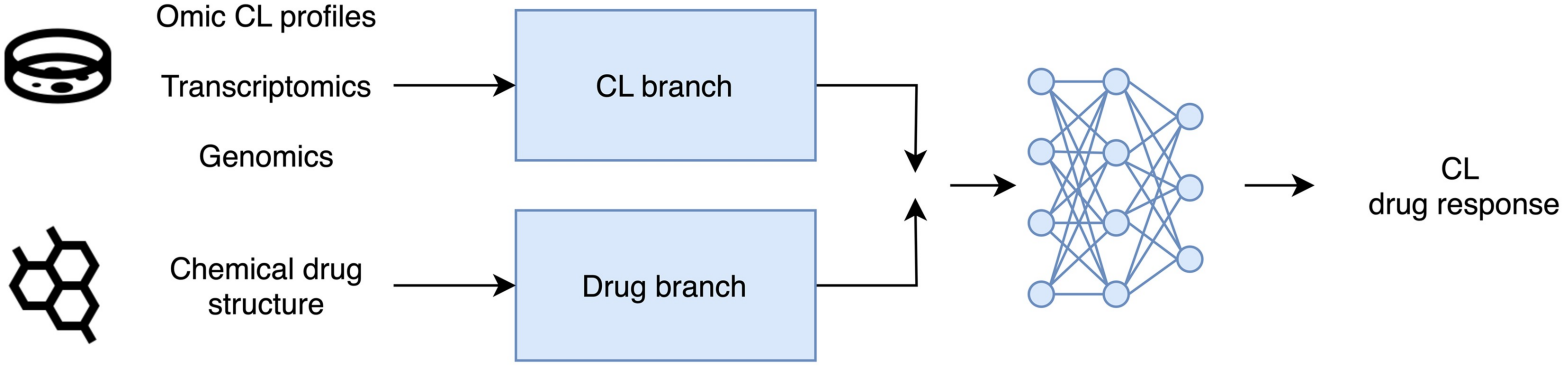
The general architecture of the models considered in this paper. Separate branches are used to encode the omics profiles and drug structures before being combined and fed through an MLP.

The availability of such datasets has resulted in a surge in new deep learning (DL) methods for predicting drug efficacy (Gerdes *et al.* 2021, Jiang *et al.* 2022, Liu *et al.* 2022, Nguyen *et al.* 2022, Hao et al. 2023, Partin *et al.* 2023, Gong *et al.* 2024, Shubham *et al.* 2024). The dominant paradigm for DRP models is to take omics profiles of cancer cell lines and drug structures as inputs to predict drug response. Such models have been reported to show state-of-the-art performance (Jiang *et al.* 2022, Liu *et al.* 2022, Nguyen *et al.* 2022, Shubham *et al.* 2024). See Partin *et al.* (2023) for a review of DL methods in DRP. DRP models typically feed each of the two inputs (cell line and drug) through separate branches, to encode these inputs. They then use the fused encoded representations to predict how effective the input drug is for the input cell line. Figure 1 shows a diagrammatic representation of this. Earlier DRP models used the same subnetwork architectures for the drug and cell line branches, e.g. tCNNS used two subnetworks made from convolutional layers (Liu *et al.* 2019). More recent models have used different subnetworks for the branches to leverage the different modalities of the input data.

Typically DRP models have used convolutional or dense layers for the cell line branches (Liu *et al.* 2019, 2020, Kim *et al.* 2021, Partin *et al.* 2021, Jiang *et al.* 2022, Liu *et al.* 2022, Nguyen *et al.* 2022, Xia *et al.* 2022, Branson *et al.* 2024). In addition to these layers, there are DRP models that use transformers (Jiang *et al.* 2022), and graph convolutional layers (Liu *et al.* 2020, Kim *et al.* 2021, Nguyen *et al.* 2022) for their drug branches. There is a large variation in the architectures of the drug branches because drugs are three-dimensional chemical substances made of atoms bonded together. Therefore, there are many different ways to both numerically represent drugs and extract features from these representations. Due to the above, there are multiple differences in the representation of data and network structures of DRP models. Thus, when a new model outperforms an old one it is not clear if the improvement comes from enhancements to the model’s architecture, a better representation of the input data or a combination of the two. To compound this problem, DRP models are normally trained and tested in three different ways corresponding to the three different aims of DRP and it is standard for only one testing type to be used for ablation studies. Furthermore, the results and metrics can be stratified in two different ways, which we will show can also significantly impact the reported performance. However, this has been overlooked in previous studies.

Thus, in this paper, we look at how the different data types and subnetworks of three models, with reported state-of-the-art performance, impact their performance for DRP for the three testing types. These testing types are (i) mixed-set (ii) cancer blind, and (iii) drug blind testing where evaluation is done using unseen drug cell line pairs, cell lines and drugs, respectively. The three models we use are tCNNS (Liu *et al.* 2019), DeepTTA (Jiang *et al.* 2022), and GraphDRP (Nguyen *et al.* 2022). Each of these models uses different drug branch architectures. tCNNS uses convolutional layers, DeepTTA uses a transformer and GraphDRP uses graph convolutional layers. We also introduce three simple but effective null hypothesis baselines, one for each testing type. These baselines do not use any omics data or drug structures. The baselines test how much performance improvement using omics data and drug structures adds and show how much performance can be attributed to patterns in the training truth values. This study shows that, for multiple testing types and models, all or most of the performance can be attributed to patterns in the training truth values. Furthermore, we find that, for multiple testing types, no performance comes from input chemical drug structures and instead, performance is due to the transcriptomics profiles.

For all of the above the models are trained and tested using continuous response values, as was done for the original implementations of these models (Liu *et al.* 2019, Jiang *et al.* 2022, Nguyen *et al.* 2022); and is typical for DRP (Partin *et al.* 2021). However, there is significant experimental noise in these response values due to the complex nature of the wet lab experiments (Haibe-Kains *et al.* 2013, Safikhani *et al.* 2016). Thus, we hypothesize that the models are overfitting to the experimental noise in these values rather than learning chemically relevant features that are predictive of drug sensitivity and that, by binarizing these response values, to remove some of the experimental noise the models can instead learn useful chemical features, resulting in better performance.

We test this by creating two transformer-based models BinaryET (encoder transformer), and BinaryCB (binary ChemBERTa) that predict binary drug response values. BinaryCB uses the chemical foundation model ChemBERTa (Chithrananda *et al.* 2020, Ahmad *et al.* 2022), for its drug branch. ChemBERTa is pre-trained using a large corpus of SMILES strings and has a BERT-like architecture (Devlin *et al.* 2019). In contrast, we train BinaryET from scratch. We then conduct ablation studies on BinaryET and BinaryCB, removing the drug branch to show the models learn useful drug features. To our knowledge, this is the first time chemical drug structures have been shown to improve performance across multiple DRP testing types. Comparing BinaryET with BinaryCB shows that the chemical foundational model does not improve performance. Re-training DeepTTA with binarized truth values we find that it can also learn useful chemical drug features, showing that binarizing the drug response values is what causes the models to learn useful drug features. Furthermore, we show that BinaryET improves performance over the published models that report state-of-the-art performance.

## 2 Methods

To make the definition of DRP more concrete consider $x_{i}^{c}$ and $x_{j}^{d}$ representation of the $i^{th}$ cell line and $j^{th}$ drug respectively and associated truth value $y_{i,j}$. Here $y_{i,j}$ is the drug response value associated with the (i,j) cell line drug pair, which describes how effective the $j^{th}$ drug is at killing the $i^{th}$ cell line. For example, *y* could be an IC50 value, the concentration of a drug needed to reduce the activity of a cell line by 50%.

A DRP model, *M* created by a learning algorithm takes $x_{i}^{c}$ and $x_{j}^{d}$ as inputs and predicts for the corresponding truth value ${\hat{y}}_{i,j}$ such that $M(x_{i}^{c},x_{j}^{d})={\hat{y}}_{i,j}$.

### 2.1 Evaluating DRP models

The aim of drug response prediction is to find a model using *T* that performs well on unseen data. This can be done by evaluating the model on some held-out testing data *E*. There are three common ways of constructing *T* and *E* corresponding to the three different objectives for DRP as outlined in the introduction.

1. Mixed-set: any drug and any cell line can be in either *T* or *E*, but a drug cell line pair can only be in one set.
2. Cancer blind: cell lines in *T* cannot be in *E*, but all drugs are in both sets.
3. Drug blind: drugs in *T* cannot be in *E*, but all cell lines are in both sets.

**Figure 2.**
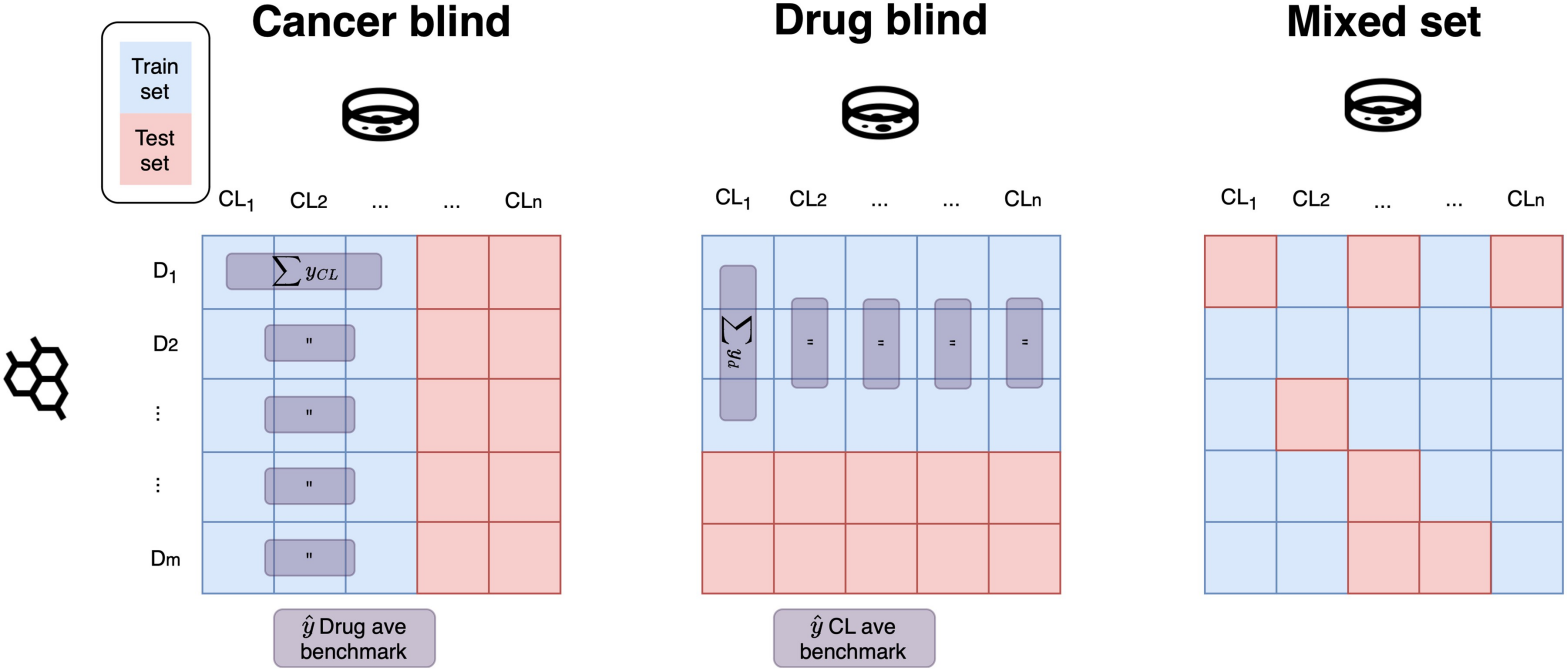
The different ways of splitting response values. Each square represents the efficacy of drug *j* for cell line *i*. The blue and red squares represent training and testing response values, respectively. The input drug and cell line representations are also split into the appropriate set, given by the response. The purple boxes represent how the predictions for the null baselines introduced in section 2.4 are calculated.

Figure 2 diagrammatically shows these different splits.

Using mixed-set testing shows if the model could be used for drug repurposing. This is because, for a given dataset a cell line in this dataset is typically not evaluated against all drugs in the dataset. Therefore, the model can be used to evaluate drug cell line pairs that have not yet been explored. This is a quick and inexpensive way to predict if a known drug (drug in the training set) can be repurposed for a known cancer sub-type. Cancer blind testing shows if the model could be used to find candidate drugs that might be effective for cancer sub-types, not in the training set. This would be useful in drug discovery, where cancer cell lines can be used to narrow down candidate drugs. Furthermore, cancer blind testing is a suitable method for simulating how good a model would be in a stratified medicine context. Where the model would need to predict efficacy for samples it was not trained on. For example to predict if a drug, or set of drugs, is suitable for new patients. A model that performs well using drug blind testing could be used to find novel anti-cancer drugs from just its structure. This is because the model would be able to predict the responses of a candidate drug to known cell lines.

### 2.2 Stratification of results in metric calculations

There are two inputs to DRP models. Thus, metrics can be stratified in two different ways, by cell line or by drug. We consider cancer blind testing in the following to make our examples concrete. However it is important to note that metrics for drug blind testing can be stratified in the analogous way. We define stratifying results by cell line as finding a metric for each cell line in the test set individually, across all drugs, before averaging the results. Similarly, we define stratifying the results by drug as finding a metric for each drug, across all cell lines in the test set, before averaging the results. In contrast, a non-stratified metric is found once across all cell line drug pairs in the testing set without averaging. Thus, there are three different ways performance metrics can be reported. In DRP studies this is typically not discussed and only stratification by cell line is reported (Jiang *et al.* 2022, Liu *et al.* 2022, Nguyen *et al.* 2022, Partin *et al.* 2023). However, as we will show, how results are stratified makes a big impact on cancer blind testing results.

The two different methods for stratification correspond to evaluating the model for distinct problems the model could be applied to. Stratifying by cell line corresponds to simulating the performance in a clinical stratified medicine context which aims to recommend a drug out of all the drugs the model is trained on, for a new patient. A model that performs well using this testing would also be directly useful in a drug discovery context where cell lines are used to narrow down candidate drugs. Specifically, the model could be used, to rank treatments from the set of all drugs the model has been trained on for an unseen cancer sub-type.

In contrast, stratifying by drug corresponds to simulating the performance in a clinical stratified medicine context where there is a set of new patients and you want to recommend if a given drug should be taken for each of these patients. This is typically the scenario in which DRP models are applied to (Gerdes *et al.* 2021, Sharifi-Noghabi *et al.* 2021a, He *et al.* 2022, Shubham *et al.* 2024). The corresponding application in a drug discovery context directly using cell lines, is where you have a candidate drug that has already been screened for some cell lines and you can then use the model to screen a set of many unseen cancer sub-types for efficacy. We provide the equations for calculating the stratified metrics in Supplementary Appendix S1.

### 2.3 Datasets and models

At a high level, our model, BinaryET consists of three subnetworks: a drug and cell line branch and a regressor, as shown in Fig. 1. Each branch separately encodes the input data before the outputs of the branches are concatenated and passed through a regressor. For our cell line branch, we used all the transcriptomic features for our cell line profiles as input to a three-layer multilayer perceptron (MLP). For our drug branch, we used SMILES strings tokenized with the ChemBERTa tokenizer (Chithrananda *et al.* 2020). We then used the tokenized SMILES strings as input to a stack of transformer encoder layers (Vaswani *et al.* 2017). We then passed the concatenated outputs of the two branches through a 3-layer MLP, that predicted the binarized response value of the input drug cell line pair. For BinaryCB we replaced the drug branch of BinaryET with ChemBERTa and fine-tuned the whole model during training. For further details of the datasets used and how we binarized the response IC50 values see Supplementary Appendix S3. For model hyperparameters see Supplementary Appendix S6.

An 80%, 10%, 10% train, validation, and test split was used for all testing and the validation data was used to select hyperparameters and the optimal number of epochs. We ran the models for three train test splits and for each split, we ran the model for three different seeds. We also repeated this for each of the testing types, mixed-set, cancer blind and drug blind. This same training protocol was also used for the published models that we recreated. See Supplementary Appendix S4 for further details on how we recreated these models to predict both continuous and binary response values.

### 2.4 Null hypothesis baselines

We create three null hypothesis baselines, (i) drug average (ii) cell line (CL) average and (iii) marker baseline, for use in cancer blind, drug blind, and mixed-set testing, respectively. None of these baselines use any omics data or drug structures. We note that the drug and CL average baselines have recently been shown to have strong performance (Li *et al.* 2023, Branson *et al.* 2024) but have not yet been fully explored. The baselines are defined as follows:

#### 2.4.1 Drug average baseline

Predictions for response values for a given drug in the test set were simply calculated as the mean of all response values for that drug in the training set. This is shown in Fig. 2 under the cancer blind split. The figure shows that for a given drug, a row in the figure, the average is calculated across cell lines in the training set (the boxes in blue). This average is then the prediction for the cell line drug pairs in the test set for that drug (the red boxes). Thus, for the drug average baseline the prediction for the drug, *d*, and any cell line, $c,\in C_{\mathrm{test}}$, ${\hat{y}}_{dc_{\mathrm{test}}}$ is given by


$${\hat{y}}_{dc_{\mathrm{test}}}=\frac{\sum_{c\in C_{\mathrm{train}}}y_{dc}}{n_{dC_{\mathrm{train}}}}.$$

The sum runs over all cell lines in the training set $c\in C_{\mathrm{train}}$ where drug cell line pair (*d*, *c*) has an associated response value $y_{dc}$. $n_{dC_{\mathrm{train}}}$ is the number of drug cell line pairs in the training set that include *d*. Note that due to missing response values $n_{dC_{\mathrm{train}}}$ can be different for different drugs.

#### 2.4.2 CL average baseline

The above was done with cell lines instead of drugs. Thus, the prediction for the cell line, *c* and any drug in the testing set $d\in D_{\mathrm{test}}$ is given by


$${\hat{y}}_{d_{\mathrm{test}}c}=\frac{\sum_{d\in D_{\mathrm{train}}}y_{dc}}{n_{cD_{\mathrm{train}}}}.$$

Where $n_{D_{\mathrm{train}}c}$ is the number of drug cell line pairs in the training set that include *c*. Similarly Fig. 2 shows the predictions of the CL average baseline under the drug blind split.

#### 2.4.3 Marker baseline

We define a marker representation of either a cell line or drug to mean a vector that uniquely identifies that cell line or drug but doesn’t have any biological or chemical properties. Here, we use a column vector to achieve this, by one-hot encoding each drug or cell line. The marker baseline took cell line and drug inputs as marker representations and fed them through an MLP.

We hypothesized each baselines were effective for similar reasons:

- Drug average baseline: there are drugs that are generally effective at killing most cell lines and other drugs that are ineffective for most cell lines.
- CL average baseline: there are cell lines that are generally harder or easier to kill for most drugs.
- Marker baseline: all drugs and cell lines are in the training set for mixed-set evaluation. Thus, a combination of the above properties can be learnt during training for inference.

Importantly, all of the null hypothesis baselines allowed us to see if adding omics data or drug structures improves performance and how much of model performance can be attributed to the above property of the data.

## 3 Results and discussion

### 3.1 Cancer blind testing with continuous drug response values

**Figure 3.**
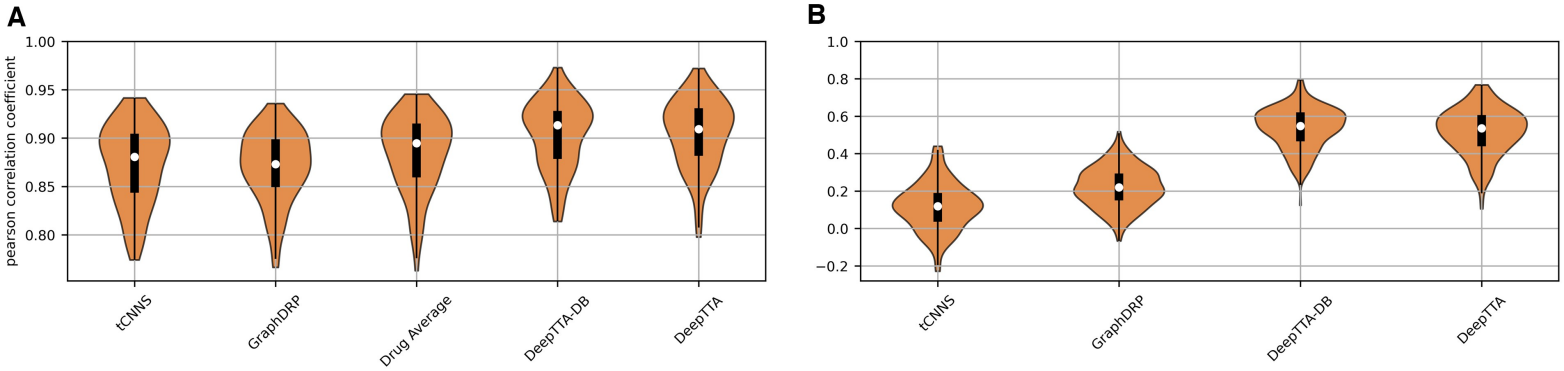
Model performance for cancer blind testing evaluated using (A) stratification of response values by cell line and (B) cancer blind stratification of response values by drug. (A) shows the distribution of performance for each cell line in the test set across all drugs. (B) shows the distribution of performance for each drug across all cell lines in the test set. The drug average baseline is not shown in (B) as it has an undefined Pearson correlation for drug stratification. Results are shown for the first model seed.

Table 1 shows the results of cancer blind testing for the published model’s and the drug average baseline and DeepTTA-DB. See Supplementary Appendix S2 for details of the metrics we report. Figure 3 shows the distribution of model performance on a per drug and per cell line basis. The reported performance difference seen between the stratified and non-stratified results (defined in Section 2.2) shows that cell line stratification is an easier problem. This is expected and is due to the statistics of the response values specifically due to observation 1:

**Table 1.** Metrics for cell line and drug stratified cancer blind testing, for three models from the literature: tCNNS, DeepTTA, GraphDRP, DeepTTA-DB (marker representation of drugs rather than transformer drug branch), and our null hypothesis drug average baseline.^a^

Method	MSE CL strat	Pear CL strat	$R^{2}$ CL strat	Pear drug strat	$R^{2}$ drug strat
tCNNS	2.41 ± 0.04	0.870 ± 0.003	0.61 ± 0.01	0.16 ± 0.02	−0.21 ± 0.01
GraphDRP	2.41 ± 0.08	0.872 ± 0.004	0.60 ± 0.01	0.220 ± 0.009	−0.26 ± 0.06
Drug average	2.190	0.884	0.640	N/A	−0.012
DeepTTA-DB	1.575 ± 0.009	0.902 ± 0.001	**0.743 ± 0.004**	**0.526 ± 0.009**	**0.257 ± 0.004**
DeepTTA	**1.57 ± 0.02**	**0.904 ± 0.002**	**0.743 ± 0.005**	0.523 ± 0.006	0.253 ± 0.008

a See Supplementary Appendix S2 for details of the metrics we report. The best model performance per metric is highlighted in **bold** for this and the following tables. The uncertainties in this and all following tables are the standard deviations across three model seeds for a given train test split.

Observation 1. Much of the variance in the response values between drugs can be explained by the average behaviour of the drugs.

We note that Observation 1 is a direct implication of the hypothesis made in Section 2.4, that there are drugs that are generally effective or ineffective. The drug average baseline clearly demonstrates Observation 1 as it is directly built to reflect this property. Thus, when we test across all of the drugs, Observation 1 boosts the performance of the models. In contrast with drug stratification where performance is evaluated for a given drug, the variance cannot be explained by Observation 1 as we are looking across cell lines for that drug. This is again shown by the drug average baseline that predicts the same value, for a given drug across all cell lines, and thus, gives an $R^{2}∼0$ and has an undefined Pearson correlation coefficient. This is also why the models have a much better performance differential compared with the baseline, for drug than cell line stratification. This shows the importance of considering how results are stratified, whereas DRP studies typically only report cell line stratified metrics and to our knowledge do not consider drug stratification. Furthermore, it shows that deep learning models can have the most impact for a drug stratified use case. Interestingly, previous work using learning curves found that the performance of the drug average baseline plateaued at larger dataset sizes but models using omics data did not. This suggests that the performance differential to the baseline will increase as more data is collected (Branson *et al.* 2024).

We repeated the above analysis for two further test train splits. The values of all metrics are similar to those in Table 1 and the average of the results over the multiple splits are shown in Fig. S5 in the Supplementary Appendix S7. Furthermore, the results agree with the conclusions arrived at in the above discussion. The results tables for the two other test train splits are shown in Supplementary Appendix S7.

**Figure 4.**
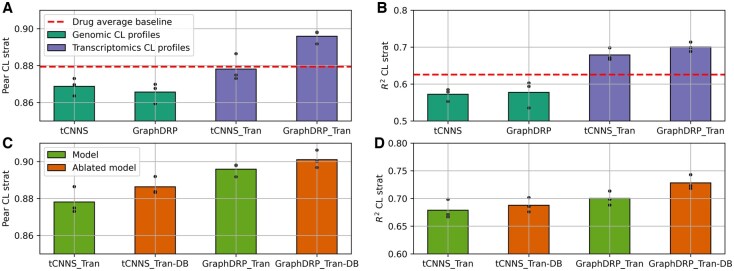
Performance of the models from the literature and variants we create averaged over all three train test splits. The top rows (A, B) compare the original models (tCNNS and GraphDRP) with the ones we created (tCNNS_Tran and GraphDRP_Tran) by adding transcriptomics profiles. The plots show that adding transcriptomics profiles causes the models to outperform the baseline. The bottom row (C, D) compares the ablations and shows the models do not learn predictive features from their chemical drug structures.

Table 1 also shows the drug average baseline outperforms both of the models that use genomic cell line profiles, tCNNS and GraphDRP, in terms of all metrics bar drug stratified Pearson correlation. Thus, these models are not generally learning useful features above those that can be derived from Observation 1. On the other hand, the two models that use transcriptomic cell line profiles, DeepTTA and DeepTTA-DB comfortably outperform the baseline for all metrics. This suggests that transcriptomic profiles play a key role in cancer blind testing. To further explore this result we constructed tCNNS_Tran and GraphDRP_Tran, where we replaced the genomic cell line profiles of tCNNS and GraphDRP with transcriptomic profiles with all genes as features. The results for these models are shown in Fig. 4 and in Table S13 in Supplementary Appendix S7. The results shows that tCNNS_Tran and GraphDRP_Tran both outperform the baseline further showing that the strong performance is caused by the transcriptomic cell line profiles. There is also a strong theoretical justification for this result. Many of the processes in cancer cannot be explained by genomic profiles (mutation and copy number variation data) alone (Lin and Yang 2019). In contrast, transcriptomics operates at the level of post-transcription so contains vital information about biochemical pathways, important for understanding diseases, that genomics does not (Khodadadian *et al.* 2020). Therefore, transcriptomics describes parts of the biology of cancer that genomics cannot.

Table 1 also shows the results for DeepTTA minus drug branch (DeepTTA-DB). We created DeepTTA-DB to see how much impact chemical drug structures have on cancer blind performance. For DeepTTA-DB instead of DeepTTA’s transformer drug branch, we simply feed in a one-hot encoded marker representation of the drugs. The table shows that DeepTTA-DB and DeepTTA perform equally within the margin of uncertainty. Therefore, the performance is due to the omics branch, further confirming that the transcriptomic cell line profiles cause the performance improvement seen over the drug average baseline. We further explore this result by removing the drug branch from tCNNS_Tran and GraphDRP_Tran to create tCNNS_Tran-DB and GraphDRP_Tran-DB. As with DeepTTA-DB, tCNNS_Tran-DB and GraphDRP_Tran-DB are tCNNS_Tran and GraphDRP_Tran with a one-hot encoded marker representation of the drugs used in place of the respective drug branches. The results for these models are shown in Fig. 4 and Table S13 in Supplementary Appendix S7. The results show that the drug branches do not improve model performance. This supports the above discussion, that the performance improvements over the baseline are due to the transcriptomic cell line profiles and the models do not learn useful representations from the chemical drug structures. Important future directions would be to evaluate if this finding is seen across datasets and if model architectures not considered here can be leveraged to learn useful chemical drug features.

We note that it is expected that for cancer blind testing transcriptomics profiles will contribute more than chemical drug profiles. This is because, in cancer blind testing, all drugs are in both the training and testing set. Therefore, the model can learn the distribution of the drug’s efficacy during training and directly use this information in the test set. This is demonstrated by the drug average baseline that shows good performance, on the test set, only using the distribution of the drug efficacy in the train set. In contrast, the cell lines tested on are unseen, therefore the omics profiles are required for the model to discriminate on. This is because, for a given drug, there is no other way for the model to differentiate its prediction than with the cell line profiles.

### 3.2 Mixed-set and drug blind testing with continuous drug response values

Table 2 shows the results of mixed-set testing for the three published models and the marker baseline. It shows that none of the published models outperform the marker baseline, for all metrics. The marker baseline does not use omics data or chemical drug features. This means the performance of the model comes from the truth values in the training set. Therefore, the published models are unable to use omics or drug data to improve performance over what information can be gained from the truth values in the training set. The marker baseline is so successful because, in mixed-set testing, every drug and cell line in the test set is also in the training set while unique drug cell line pairs are only in the training or testing set. Therefore, the model has ample information on the distribution of response values for any cell line or drug in the training set to learn from. Thus, rather than having to learn important cell lines or drug features, it can just use these distributions for predictions. We repeated the above analysis for two further test train splits to increase the reliability of our results. The results for these splits support Table 2 and are shown in Supplementary Appendix S8. We also note that DRP studies typically do their ablation studies for this testing type, limiting their usefulness in light of the above.

**Table 2.** Metrics for mixed-set testing for three models from the literature and our null hypothesis marker baseline (marker).^a^

Method	MSE	Pear	$R^{2}$
tCNNS	1.256 ± 0.004	0.9104 ± 0.0003	0.8283 ± 0.0005
GraphDRP	1.06 ± 0.04	0.930 ± 0.001	0.856 ± 0.005
DeepTTA	0.98 ± 0.01	0.931 ± 0.001	0.866 ± 0.002
Marker	**0.88 ± 0.01**	**0.9379 ± 0.0007**	**0.880 ± 0.001**

^a^ The published models do not outperform the baseline for any metric. Bold values indicate best performance.

To further investigate this result we created marker versions of each of the literature models. We did this by replacing the drug branches and omics inputs of the models with a marker representation before re-training and testing the models. The results given in Supplementary Appendix S8 show that all marker versions of the models outperform the original models. This supports the hypothesis that the models are learning the distribution of efficacy/susceptibility of drugs/cell lines by associating the IC50 values with a marker representation of the inputs, instead of learning biological or chemically relevant features from the input omics or chemical structures and removing the omics/chemical input features allows the model to more easily do this. Therefore, the reason for the improved performance is the removal of omics/chemical features with marker representations. These results show the importance of using marker representations to baseline DRP models for mixed-set testing.

For drug blind testing we found a much greater variation in performance between stratified and non-stratified testing than we found for cancer blind testing. There was also a greater variation between different train test splits than we found for the other testing types. The results for all splits, stratified and non-stratified testing are shown in Supplementary Appendix S9. These results show that while there are large differences between the test train splits, for each split there are metrics that outperform the CL average baseline. Therefore, the omics and drug structures can provide a benefit for non-stratified drug blind testing. However, more needs to be done to improve the model’s drug blind testing abilities so that they outperform the baseline consistently by improving model stability.

### 3.3 Evaluation of BinaryET

Table 3 shows the results for drug stratified cancer blind testing using binary response values. It shows our model, BinaryET outperforms all other models that had previously reported state-of-the-art performance, across all metrics. All models outperform the drug average baseline for the area under the curve (AUC) and area under the precision-recall curve (AUPR). We note that by definition the drug average baseline has an AUC of 0.5, as is seen in Table 3. The results for two further train test splits are shown in Supplementary Appendix S10, they support the results in Table 3. Supplementary Appendix S10 also shows the metrics for the same set of results but with cell line stratification and without stratification. It supports the result from Table 3 that BinaryET outperforms all other models. However, unlike in Table 3 the drug average baseline outperforms GraphDRP and tCNNS for both AUC and AUPR. These results also support the difference between cell line and drug stratified metrics seen for continuous response values.

**Table 3.** Metrics for drug stratified drug blind testing for BinaryET (ours) compared with the published models, retrained to predict binary response values, and the null hypothesis drug average baseline.^a^

Method	AUC	AUPR
Drug average	0.500	0.320
tCNNS	0.598 ± 0.008	0.40 ± 0.01
GraphDRP	0.6173 ± 0.0009	0.421 ± 0.007
DeepTTA	0.747 ± 0.008	0.53 ± 0.02
BinaryET	**0.771 ± 0.003**	**0.569 ± 0.004**

^a^ As in the previous and following tables, the uncertainties are the standard deviations across three model seeds for a given train test split. Bold values indicate best performance.

Tables 4 and 5 show the results for the ablation study where we remove the drug branch from BinaryET. The table shows for all splits and ways of stratifying the results removing the drug branches decreases the performance of our model. Therefore, BinaryET is able to extract useful representations from the chemical drug structures. Supplementary Appendix S10 shows this result also holds for DeepTTA. This is in contrast to the results we found when continuous truth values are used (Table 1). Thus, binarizing the truth values allows the models to successfully leverage chemical drug structures. We hypothesize that this is because due to the experimental noise in the continuous response values, they do not fully reflect the underlying causal biochemistry of the experiment. Thus, there are not chemically relevant features that are predictive of continuous drug sensitivity that the models can learn, over what can be learnt from the transcriptomics. Then, binarizing these response values removes some of the experimental noise better aligning the binary response values with underlying biochemistry, which the input chemical drug structures can explain allowing models to learn useful features from these inputs.

**Table 4.** Ablation study for BinaryET removing the drug branch BinaryET-DB, for all types of stratified cancer blind testing.^a^

Stratification type	BinaryET AUC	BinaryET-DB AUC
Drug Strat	**0.771 ± 0.003**	0.76 ± 0.01
CL Strat	**0.9305 ± 0.0006**	0.928 ± 0.001
No Strat	**0.9158 ± 0.0006**	0.913 ± 0.001

a Bold values indicate best performance.

**Table 5.** Ablation study for BinaryET removing the drug branch BinaryET-DB, for all types of stratified cancer blind testing.^a^

Stratification type	BinaryET AUPR	BinaryET-DB AUPR
Drug Strat	**0.569 ± 0.004**	0.55 ± 0.02
CL Strat	**0.822 ± 0.002**	0.817 ± 0.001
No Strat	**0.8084 ± 0.0002**	0.8033 ± 0.0008

a Bold values indicate best performance.

Table 6 shows the results for BinaryCB, where we have replaced the drug branch in BinaryET with ChemBERTa. We have evaluated different versions of ChemBERTa pre-trained using databases of SMILES strings of different sizes, from 40 000 SMILES strings from the ZINC database to 77 000 000 from the PubChem database. The table also shows the results for an ablation of BinaryCB, Marker DB where we replace the drug branch with a marker drug input. The table also includes results for a model with the same architecture and hyperparameters as BinaryCB but without any pre-training (No PT) which is the same as BinaryET with different hyperparameters. The tables show that while BinaryCB does learn useful chemical drug features, the pre-training does not provide any performance improvements over training a model from scratch. The table also shows that increasing the pre-training size of ChemBERTa does not improve performance. Furthermore, moving from ZINC to PubChem leads to a drop in performance despite an order of magnitude increase in the number of SMILES strings used for pre-training. A possible reason for this is that the distribution of SMILES strings taken to train ChemBERTa from PubChem may be further away from anti-cancer drugs than those that were chosen from ZINC.

**Table 6.** Performance of BinaryCB with different versions of ChemBERTa used for the drug branch.^a^

	BCE (Loss)	AUC	AUPR
zinc40k	0.3448 ± 0.0003	0.9169 ± 0.0002	0.8561 ± 0.0002
zinc100k	0.345 ± 0.001	0.9170 ± 0.0006	0.8558 ± 0.0006
zinc250k	0.3432 ± 0.0006	0.9169 ± 0.0002	0.8564 ± 0.0003
Pub5M	0.354 ± 0.001	0.9140 ± 0.0005	0.8512 ± 0.0005
Pub10M	0.357 ± 0.003	0.914 ± 0.002	0.851 ± 0.002
Pub77M	0.360 ± 0.001	0.9123 ± 0.0006	0.849 ± 0.001
Marker DB	0.3502 ± 0.0007	0.9136 ± 0.0001	0.8506 ± 0.0005
No PT	0.3429 ± 0.0007	0.917 ± 0.001	0.857 ± 0.001
BinaryET	**0.342 ± 0.002**	**0.919 ± 0.001**	**0.858 ± 0.002**

a Performance is the average of three model seeds on the validation set. Bold values indicate best performance.

Table 7 shows the results for mixed-set testing with binary response values. It shows that our model, BinaryET, outperforms all other models that had previously reported state-of-the-art performance. In contrast to the continuous case, all models outperform the null hypothesis baseline. However, it still performs comparatively to the models suggesting that much of the performance is due to patterns directly inferred from the training truth values. Furthermore, the table shows that removing the drug branch from BinaryET decreases the performance. Thus, BinaryET also learns useful information from its drug branch for mixed-set in addition to cancer blind testing. The results for two further train test splits are shown in Supplementary Appendix S11, they agree with the results in Table 7.

**Table 7.** Metrics for mixed-set testing for BinaryET (ours) compared with the published models, retrained to predict binary response values, and the null hypothesis marker baseline.^a^

	AUC	AUPR
Marker baseline	0.9276 ± 0.0001	0.8634 ± 0.0001
tCNNS	0.928 ± 0.001	0.863 ± 0.001
GraphDRP	0.9376 ± 0.0007	0.882 ± 0.001
DeepTTA	0.9435 ± 0.0008	0.892 ± 0.001
BinaryET-DB	0.9458 ± 0.0006	0.896 ± 0.001
BinaryET	**0.9470 ± 0.0004**	**0.8983 ± 0.0008**

a Bold values indicate best performance.

## 4 Conclusion

In this paper, we recreated three drug response prediction (DRP) models, that have reported state-of-the-art performance. We also created null hypothesis baselines that did not use omics data or chemical features to help understand the published model’s sources of performance. These baselines showed strong performance relative to the models suggesting that they should be used when evaluating future DRP research. They also revealed that for multiple testing types, the performance could partially or fully be explained by patterns in the training truth values. For cancer blind testing we found, using ablation studies, that none of the model performance comes from their chemical drug structures, instead, it is due to the transcriptomics cell line profiles. To address these limitations we created BinaryET and BinaryCB, to predict binary drug response values guided by the hypothesis that this will remove some of the experimental noise allowing them to learn useful chemical features. We find this to be the case for multiple testing types. Furthermore, we show BinaryET improves upon the performance of the models that have reported state-of-the-art performance.

## Author contributions

Nikhil Branson (Conceptualization, Data curation, Formal analysis, Funding acquisition, Investigation, Methodology, Project administration, Software, Validation, Visualization, Writing—original draft, Writing—review & editing), Pedro R. Cutillas (Supervision, Writing—review & editing), and Conrad Bessant (Conceptualization, Funding acquisition, Project administration, Resources, Supervision, Writing—review & editing)

## Supplementary Material

btaf255_Supplementary_Data

## Data Availability

Source code is available at https://github.com/Nik-BB/Understanding_DRP_models. The datasets needed to re-train the models are publicly available. Transcriptomics and genomics data can be downloaded from the Genomics of drug sensitivity in cancer database https://www.cancerrxgene.org/. Drug response data in the form of IC50 values can also be downloaded form values from GDSC https://www.cancerrxgene.org/ GDSC2 IC50 values are used for this study. PubChem ID’s and smiles strings for the drugs in GDSC can be found from https://pubchem.ncbi.nlm.nih.gov/.
