# Supplementary material for: Understanding the sources of performance in deep drug response models reveals insights and improvements
Source: Bioinformatics. 2025 Jul 15;41(Suppl 1):i142–9. doi: 10.1093/bioinformatics/btaf255 (PMC12261491; doi:10.1093/bioinformatics/btaf255)
Supplement: btaf255_Supplementary_Data [file btaf255_supplementary_data.pdf]

Appendix / supplemental material

Appendix A. Calculating stratified metrics

For a cell line stratified metric MC_{strat},

$$MC_{strat} = \frac{\sum_{c \in C} M_c}{N_c}. \tag{1}$$

Where the sum runs over, C all cell lines in the test set and N_c is the number of cell lines in the test set. M_c is the metric M for cell line c such that

$$M_c = f(y_c, \hat{y_c}).$$

Where y_c and $\hat{y_c}$ are the truth values and predicted values for the cell line drug pairs that include c respectively. f is a function that gives metric M for example it could be the mean squared error. In contrast drug-stratified metric is instead given by MD_{strat},

$$MD_{strat} = \frac{\sum_{d \in D} M_d}{N_d} \tag{2}$$

Where the sum runs over, D all drugs and N_d is the number of drugs. M_d is the metric M for drug d such that

$$M_d = f(y_d, \hat{y_d}).$$

Where y_d and $\hat{y_d}$ are the truth values and predicted values for all drug cell lines pairs that include d , and are in the test set.

Similarly, drug stratified drug blind testing is defined by equation 2 but with the sum only running over drugs in the testing set, and N_d giving the number of drugs in the testing set. Furthermore, cell line stratified drug blind testing is defined by equation 1 but with the sum running over all cell lines, and where y_c, $\hat{y_c}$ are only the truth and predicted values for the drug cell line pairs in the test set that contain c.

A non-stratified M is simply given by

$$M = f(y, \hat{y}).$$

Where y and $\hat{y}$ are the truth values and predicted values for the cell line drug pairs in the test set respectively.

Appendix B. Metrics reported

When evaluating models that predicted continuous drug response values we calculate Mean squared error (MSE), Pearson correlation coefficient (Pear) and the Coefficient of determination (R^2). We report Pear and R^2 for both cell line (CL) stratification and drug stratification as well as non-stratification, as described above.

When evaluating models that predicted binary drug response values we calculate the area under the receiver operating characteristic curve (AUC) and the area under the precision-recall curve (AUPR).

Appendix C. Dataset details

Across this study, we used transcriptomic cell line profiles from the genomics of drug sensitivity in cancer database (GDSC) (Yang et al., 2012). For drug response data we used IC50 values from GDSC2, where IC50 is the standard measure of drug response and was used in the original papers for tCNNS DeepTTA and GraphDRP. These downloaded IC50 values were continuous measurements. Thus, we binarised them for the second part of our study when considering binary drug efficacy. We used the same method to binarise the IC50 values as Liu et al. (Liu et al., 2022), where a drug is considered ineffective if its IC50 value is more than the maximum concentration used during screening. We downloaded SMILES representations of the drugs from PubChem (Kim et al., 2023).

Table 8 shows the full dataset size used when training and testing the modles for both binary and continuous IC50 values. Only cell lines that had IC50 values and both genomics and transcriptomics cell line profiles were kept. Four drugs were removed when binarising IC50 values as they had multiple maximum concentration values. We note that not all drug cell line pairs have IC50 values in GDSC2. Thus, we also removed drug cell line pairs without IC50 values, hence why we have less than $163,624$ and $160,008$ drug cell line pairs for continuous and binary IC50 values respectively.

Table 8. Dataset size for binary and continuous IC50 values. An %80, %10, %10 train validation test split was used. For drug-stratified binary cancer blind splitting the number of drugs we tested was 147, 153, 148 for train test split 1, 2, and 3 respectively, due to AUC and AUPR not being defined for the drugs removed.

	Number of cell lines	Number of drugs	Number of drug cell line pairs
Continuous	904	181	147,713
Binary	904	177	144,101

Table 9. Number of positive and negative examples after data splitting for binary response values.

	S1 Negative	S1 Positive	S2 Negative	S2 Positive	S3 Negative	S3 Positive
train	79359	35910	79448	35898	79439	36013
test	10637	4047	10015	4394	10151	4230
val	9339	4809	9872	4474	9745	4523

Appendix D. Details of published models used

For genomics cell line profiles we used genomics data from the genomics of drug sensitivity in cancer database (GDSC) (Yang et al., 2012). Genomics profiles that included, genetic mutation and copy number variations information, were used to recreate tCNNS and GraphDRP. For transcriptomic profiles, we used transcriptomics data from GDSC. Transcriptomics profiles were used to recreate DeepTTA. We only kept cell lines that were in all three of the above datasets. Thus, 904 cell lines were used in the following analysis.

The omics data in GDSC has already undergone standard preprocessing. Where the transcriptomics data is preprocessed using the robust multi-array analysis algorithm (RMA) (Irizarry et al., 2003). This is the same data that was used in DeepTTA. Similarly, we directly used the binary genomics data from GDSC as was done in tCNNS and GraphDRP. Furthermore, the IC50 values provided by GDSC are natural logarithm transformed.

SMILES (simplified molecular-input line-entry system) and molecular graphs were used as the drug representations with chemical properties. SMILES were used in the tCNNS and DeepTTA models, while molecular graphs were used in the GraphDRP model. In a SMILES representation, each molecule is represented as a string of characters. In molecular graph representations of molecules/drugs, each node represents an atom in the molecule and each edge represents a bond between the atoms. Only drugs with SMILES strings were kept, leading to us using 181 drugs for the following analysis.

When retraining and testing these models with binary response values we used binary cross entropy for the loss function.

Appendix E. Hardwear

Models were trained using nvidia A100 GPUs, each model was trained on one GPU.

Appendix F. Model hyperparameters

The model hyperparameters for BinaryET are shown in table 10.

For the marker baseline, 3 dense hidden layers were for the MLP with 4096 neurons per layer, with ReLU activation, followed by an output dense layer with one node.

Table 10. BinaryET hyperparameters the first 6 parameters refer to the drug branch (Transformer encoder layers).

Hyperparameters	Value
Nb transformer (TF) encoder layers	8
Nb attetion heads	8
TF feed forward dim	2048
Embedding dimension	128
Dropout	0.01
layer_norm_eps	1e-05
Activation	relu
Nb classifier nodes layer 1	1024
Nb classifier nodes layer 2	256
Nb classifier nodes layer 3	64
Loss	binary cross entropy
Batch size	128
Peak learning rate	5e-05
Optimiser	AdamW
learning rate scheduler	cosine with warmup
epochs	100
Nb warm up learing rate steps	384

Appendix G. Cancer blind testing

This section shows the tables for additional cancer blind testing with continuous response values. Table 11 shows two additional train test splits for tCNNS, GraphDRP, DeepTTA-DB and DeepTTA.

Table 13 shows the results for tCNNS and GraphDRP but by replacing the genomics cell line profiles with transcriptomics profiles, (tCNNS_Tran an GraphDRP_Tran). It also shows these results for tCNNS_Tran-DB and GraphDRP_Tran-DB, removing the drug branch from the respective models. Where these tables show that adding transcriptomics cell line profiles causes the models to outperform the drug average baseline. Furthermore, removing the drug features does not decrease performance.

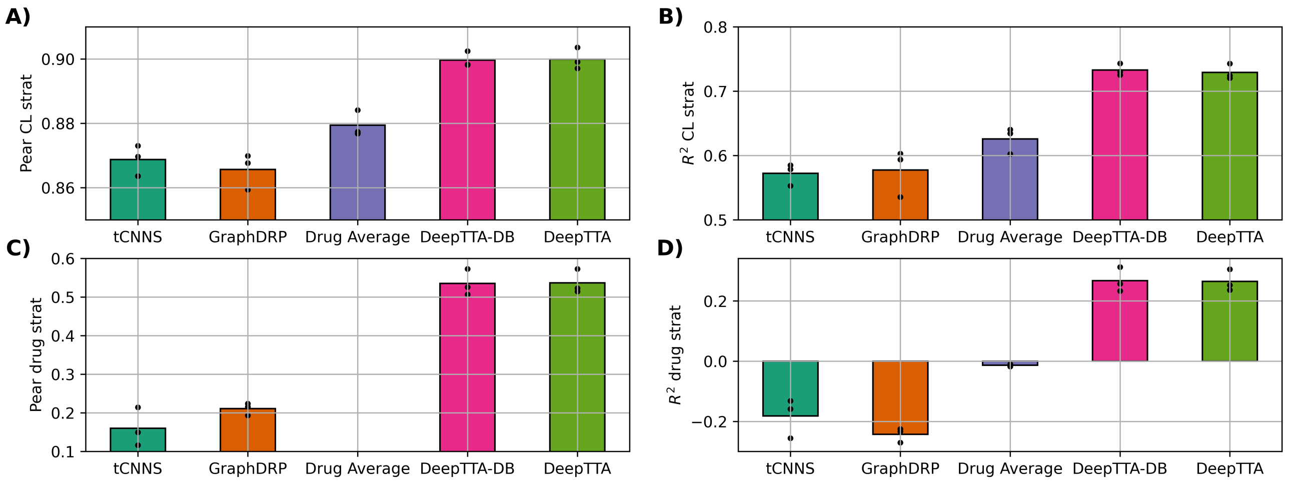

Fig. 5. Performance of the models and drug average baseline averaged over all three train test splits. Scatter points give metrics for the individual train test splits. Note that the baseline has undefined Pearson correlation (Pear) for drug stratified testing. The top row (A, B) gives the results for cell line (CL) stratification and the bottom row (C, D) for drug stratification.

Table 11. Metrics for cancer blind testing for the second and thrid test train split.

Split	Method	MSE	Pear CL strat	R^2 CL strat	Pear drug strat	R^2 drug strat	MSE drug strat
	tCNNS	2.4 ± 0.005	0.864 ± 0.002	0.620 ± 0.004	0.21 ± 0.04	-0.12 ± 0.03	2.45 ± 0.03
	GraphDRP	2.45 ± 0.05	0.867 ± 0.002	0.61 ± 0.01	0.24 ± 0.01	-0.19 ± 0.05	2.48 ± 0.04
2	Drug Average	2.303	0.877	0.634	N/A	-0.010	2.334
	DeepTTA-DB	**1.71 ± 0.02**	0.898 ± 0.001	**0.725 ± 0.004**	0.507 ± 0.007	0.23 ± 0.01	**1.73 ± 0.03**
	DeepTTA	1.72 ± 0.02	**0.8991 ± 0.0008**	0.720 ± 0.003	**0.514 ± 0.006**	**0.236 ± 0.006**	1.74 ± 0.02
	tCNNS	2.7 ± 0.1	0.865 ± 0.003	0.57 ± 0.02	0.15 ± 0.02	-0.17 ± 0.03	2.69 ± 0.07
	GraphDRP	2.7 ± 0.2	0.872 ± 0.002	0.56 ± 0.03	0.22 ± 0.02	-0.17 ± 0.09	2.7 ± 0.1
3	Drug Average	2.546	0.877	0.602	N/A	-0.018	2.505
	DeepTTA-DB	**1.679 ± 0.009**	**0.8982 ± 0.0007**	**0.7303 ± 0.0009**	**0.573 ± 0.005**	**0.312** ± 0.004	**1.676 ± 0.008**
	DeepTTA	1.70 ± 0.01	0.897 ± 0.001	0.724 ± 0.003	**0.573 ± 0.004**	0.305 ± 0.008	1.70 ± 0.01

Table 12. Cancer blind testing with no stratification (apart from last col MSE drug strat, which is only marginally different from the non-stratfied metric due to a different number of cell lines being evaluated for each drug caused by missing truth values). For three train test splits

Train test split	Method	MSE	Pear	R^2	MSE drug strat
1	tCNNs	2.42 ± 0.04	0.819 ± 0.004	0.660 ± 0.006	2.44 ± 0.03
	GraphDRP	2.42 ± 0.08	0.827 ± 0.003	0.66 ± 0.01	2.43 ± 0.08
	Drug Average	2.191	0.832	0.692	2.189
	DeepTTA-DB	**1.57 ± 0.01**	**0.883 ± 0.001**	**0.779 ± 0.002**	**1.57 ± 0.01**
	DeepTTA	**1.57 ± 0.02**	**0.883 ± 0.002**	**0.779 ± 0.003**	**1.57 ± 0.02**
2	tCNNs	2.412 ± 0.009	0.818 ± 0.002	0.664 ± 0.001	2.45 ± 0.03
	GraphDRP	2.47 ± 0.05	0.821 ± 0.002	0.657 ± 0.007	2.48 ± 0.04
	Drug Average	2.312	0.824	0.678	2.334
	DeepTTA-DB	**1.72 ± 0.02**	0.873 ± 0.001	**0.760 ± 0.003**	**1.73 ± 0.03**
	DeepTTA	1.73 ± 0.02	**0.874 ± 0.001**	0.759 ± 0.002	1.74 ± 0.02
3	tCNNs	2.7 ± 0.1	0.801 ± 0.006	0.64 ± 0.01	2.69 ± 0.07
	GraphDRP	2.7 ± 0.1	0.815 ± 0.003	0.64 ± 0.02	2.7 ± 0.1
	Drug Average	2.523	0.812	0.658	2.505
	DeepTTA-DB	**1.674 ± 0.009**	**0.880 ± 0.001**	**0.773 ± 0.001**	**1.676 ± 0.008**
	DeepTTA	1.70 ± 0.01	0.8790 ± 0.0008	0.770 ± 0.002	1.70 ± 0.01

Table 13. Cancer blind cell line stratified metrics for tCNNS and GraphDRP but by replacing the genomics cell line profiles with transcriptomics profiles, (tCNNS_Tran an GraphDRP_Tran) and for CNNS_Tran-DB and GraphDRP_Tran-DB, removing the drug branch from the respective models

Train test split	Method	MSE	Pear	R^2
1	tCNNS	2.41 ± 0.04	0.870 ± 0.003	0.61 ± 0.01
	GraphDRP	2.41 ± 0.08	0.872 ± 0.004	0.60 ± 0.01
	Drug Average	2.190	0.884	0.640
	tCNNS_Tran	1.83 ± 0.05	0.8865 ± 0.0005	0.698 ± 0.008
	tCNNS_Tran-DB	1.78 ± 0.04	0.8919 ± 0.0009	0.702 ± 0.007
	GraphDRP_Tran	1.77 ± 0.02	0.898 ± 0.002	0.714 ± 0.004
	GraphDRP_Tran-DB	1.56 ± 0.01	0.9062 ± 0.0008	0.743 ± 0.003
2	tCNNS	2.4 ± 0.005	0.864 ± 0.002	0.620 ± 0.004
	GraphDRP	2.45 ± 0.05	0.867 ± 0.002	0.61 ± 0.01
	Drug Average	2.303	0.877	0.634
	tCNNS_Tran	2.05 ± 0.05	0.875 ± 0.003	0.67 ± 0.01
	tCNNS_Tran-DB	1.94 ± 0.04	0.884 ± 0.001	0.685 ± 0.007
	GraphDRP_Tran	1.87 ± 0.06	0.898 ± 0.001	0.70 ± 0.01
	GraphDRP_Tran-DB	1.71 ± 0.03	0.9001 ± 0.0007	0.723 ± 0.004
3	tCNNS	2.7 ± 0.1	0.865 ± 0.003	0.57 ± 0.02
	GraphDRP	2.7 ± 0.2	0.872 ± 0.002	0.56 ± 0.03
	Drug Average	2.546	0.877	0.602
	tCNNS_Tran	2.07 ± 0.06	0.873 ± 0.002	0.67 ± 0.01
	tCNNS_Tran-DB	2.00 ± 0.02	0.883 ± 0.001	0.676 ± 0.005
	GraphDRP_Tran	1.93 ± 0.07	0.892 ± 0.003	0.69 ± 0.01
	GraphDRP_Tran-DB	1.75 ± 0.01	0.8968 ± 0.0006	0.718 ± 0.003

Appendix H. Mixed set testing

This section shows the tables for mixed set testing for additional train test splits for continuous drug response values. Note that all mixed set testing metrics calculated here were non stratified, so calculated once for all model predictions.

This section also shows the results for the marker versions of each of the literature models in Table 15. Here we removed the drug branches of the models and instead simply fed in a one-hot encoded marker representation of the drugs as we did when creating DeepTTA-DB. We also replaced the omics inputs of the models with a one-hot encoded marker representation before re-training and testing the models.

Table 14. Non stratified mixed set testing for train test splits 2 and 3.

Train test split	Method	MSE	Pear	R^2
2	tCNNS	1.25 ± 0.01	0.9114 ± 0.0007	0.830 ± 0.001
	GraphDRP	1.08 ± 0.02	0.9312 ± 0.0006	0.854 ± 0.003
	DeepTTA	0.973 ± 0.009	0.9317 ± 0.0008	0.868 ± 0.001
	marker baseline	**0.890 ± 0.001**	**0.9375 ± 0.0001**	**0.8789 ± 0.0002**
3	tCNNS	1.31 ± 0.01	0.9060 ± 0.0007	0.819 ± 0.002
	GraphDRP	1.047 ± 0.009	0.93 ± 0.0008	0.856 ± 0.001
	DeepTTA	1.00 ± 0.01	0.9290 ± 0.0007	0.863 ± 0.001
	marker baseline	**0.904 ± 0.006**	**0.9359 ± 0.0005**	**0.8757 ± 0.0009**

Table 15. mixed set testing with maker inputs to the literature models using the original dataset. The table shows omics and drug features do not improve model performance. **Bold** gives best metric by model type.

	tCNNS	Marker tCNNS	DeepTTA	Marker DeepTTA	GraphDRP	Marker GraphDRP
MSE	1.256 ± 0.004	**1.172 ± 0.009**	0.98 ± 0.01	**0.937 ± 0.004**	1.06 ± 0.04	**0.849 ± 0.003**
Pear	0.9104 ± 0.0003	**0.9167 ± 0.0005**	0.931 ± 0.001	**0.9341 ± 0.0003**	0.930 ± 0.001	**0.9404 ± 0.0003**
R^2	0.8283 ± 0.0005	**0.840 ± 0.001**	0.866 ± 0.002	**0.8719 ± 0.0005**	0.856 ± 0.005	**0.8840 ± 0.0004**

Appendix I. Drug blind testing

This section shows the tables for non stratified drug blind testing for all three train test splits.

Table 16. Metrics for non-stratified drug blind testing for three train test splits. The metrics in **bold** are better than, or have bounds better than, the CL average baseline.

Train test split	Method	MSE	Pear	R^2
1	GraphDRP	11.0 ± 3	-0.0 ± 0.2	-0.5 ± 0.4
	DeepTTA	7.5 ± 0.5	**0.28 ± 0.07**	-0.04 ± 0.07
	tCNNs	**7.0 ± 1**	**0.3 ± 0.1**	**0.0 ± 0.2**
	CL Average	6.493	0.318	0.093
2	GraphDRP	6.0 ± 1	**0.3 ± 0.2**	-0.1 ± 0.2
	DeepTTA	5.6 ± 0.2	**0.42 ± 0.03**	-0.02 ± 0.04
	tCNNs	6.4 ± 0.8	**0.3 ± 0.1**	-0.2 ± 0.2
	CL Average	4.999	0.348	0.090
3	GraphDRP	11.2 ± 0.8	0.06 ± 0.08	-0.27 ± 0.09
	DeepTTA	**7.7 ± 0.6**	**0.47 ± 0.01**	**0.13 ± 0.07**
	tCNNs	**6.7 ± 0.2**	**0.52 ± 0.04**	**0.24 ± 0.02**
	CL Average	8.113	0.288	0.082

Table 17. Metrics for drug stratified drug blind testing for three train test splits. The metrics in **bold** are better than, or have bounds better than, the CL average baseline. Or better than zero for the case of Pear where the CL average baseline is undefined.

Train test split	Method	MSE	Pear	R^2
1	GraphDRP	10.0 ± 3	0.552 ± 0.007	-4.0 ± 2
	DeepTTA	7.2 ± 0.5	0.55 ± 0.03	**-2.3 ± 0.3**
	tCNNs	**7.0 ± 1**	0.54 ± 0.03	**-2.3 ± 0.6**
	CL Average	6.228	0.612	-2.154
2	GraphDRP	6.0 ± 1	0.54 ± 0.01	-2.4 ± 0.6
	DeepTTA	5.4 ± 0.2	0.584 ± 0.009	-2.4 ± 0.1
	tCNNs	6.0 ± 1	0.57 ± 0.01	**-2.6 ± 0.5**
	CL Average	4.988	0.627	-2.180
3	GraphDRP	10.5 ± 0.6	0.58 ± 0.04	-4.9 ± 0.5
	DeepTTA	**8.0 ± 1**	0.61 ± 0.01	**-3.2 ± 0.5**
	tCNNs	**6.3 ± 0.2**	0.621 ± 0.001	**-1.9 ± 0.2**
	CL Average	7.633	0.659	-3.279

Table 18. Metrics for cell line stratified drug blind testing for three train test splits. The metrics in **bold** are better than, or have bounds better than, the CL average baseline.

Train test split	Method	MSE	Pear	R^2
1	GraphDRP	11.0 ± 3	-0.2 ± 0.2	**-0.7 ± 0.5**
	DeepTTA	7.5 ± 0.5	**0.1 ± 0.1**	**-0.18 ± 0.09**
	tCNNs	**7.0 ± 1**	**0.2 ± 0.2**	**-0.1 ± 0.2**
	CL Average	6.503	N/A	-0.023
2	GraphDRP	**6.0 ± 1**	**0.1 ± 0.2**	**-0.3 ± 0.3**
	DeepTTA	5.6 ± 0.2	**0.30 ± 0.04**	-0.19 ± 0.04
	tCNNs	6.4 ± 0.8	**0.1 ± 0.1**	-0.4 ± 0.2
	CL Average	5.001	N/A	-0.057
3	GraphDRP	11.2 ± 0.8	-0.1 ± 0.1	-0.4 ± 0.1
	DeepTTA	7.7 ± 0.6	**0.407 ± 0.008**	**0.04 ± 0.08**
	tCNNs	6.7 ± 0.2	**0.47 ± 0.06**	**0.17 ± 0.03**
	CL Average	8.125	N/A	-0.010

Appendix J. Binary cancer blind testing

This section shows the tables for cancer blind testing for binary response values.

Table 19. Drug and cell line (CL) stratified cancer blind testing for all three train test splits, for binary response values.

Train test Split	Method	Drug Strat AUC	Drug Strat AUPR	CL Strat AUC	CL Strat AUPR
1	Drug Average	0.500	0.320	0.916	0.801
	tCNNS	0.598 ± 0.008	0.40 ± 0.01	0.905 ± 0.001	0.771 ± 0.005
	GraphDRP	0.6173 ± 0.0009	0.421 ± 0.007	0.911 ± 0.001	0.787 ± 0.002
	DeepTTA-DB	0.739 ± 0.006	0.53 ± 0.01	0.924 ± 0.001	0.812 ± 0.002
	DeepTTA	0.747 ± 0.008	0.53 ± 0.02	0.926 ± 0.002	0.814 ± 0.003
	BinaryET-DB	0.76 ± 0.01	0.55 ± 0.02	0.928 ± 0.001	0.817 ± 0.001
	BinaryET	**0.771 ± 0.003**	**0.569 ± 0.004**	**0.9305 ± 0.0006**	**0.822 ± 0.002**
2	Drug Average	0.500	0.352	0.915	0.819
	tCNNS	0.63 ± 0.01	0.456 ± 0.002	0.9129 ± 0.0009	0.813 ± 0.002
	GraphDRP	0.631 ± 0.008	0.46 ± 0.02	0.912 ± 0.002	0.814 ± 0.004
	DeepTTA-DB	0.777 ± 0.009	0.60 ± 0.02	0.931 ± 0.003	0.840 ± 0.005
	DeepTTA	0.785 ± 0.002	**0.620 ± 0.008**	0.934 ± 0.002	0.845 ± 0.003
	BinaryET-DB	0.781 ± 0.009	0.60 ± 0.01	0.931 ± 0.003	0.839 ± 0.006
	BinaryET	**0.79 ± 0.01**	0.61 ± 0.02	**0.934 ± 0.003**	**0.846 ± 0.006**
3	Drug Average	0.500	0.339	0.917	0.817
	tCNNS	0.588 ± 0.008	0.427 ± 0.005	0.9055 ± 0.0008	0.785 ± 0.004
	GraphDRP	0.599 ± 0.008	0.43 ± 0.01	0.9109 ± 0.0009	0.803 ± 0.001
	DeepTTA-DB	0.785 ± 0.007	0.6 ± 0.008	0.9308 ± 0.0007	0.8390 ± 0.0005
	DeepTTA	0.789 ± 0.005	0.601 ± 0.003	0.932 ± 0.001	0.842 ± 0.002
	BinaryET-DB	0.795 ± 0.003	0.608 ± 0.002	0.933 ± 0.001	0.841 ± 0.004
	BinaryET	**0.798 ± 0.003**	**0.613 ± 0.005**	**0.9348 ± 0.0006**	**0.844 ± 0.001**

Table 20. Non stratified cancer blind testing

Train test split	Method	AUC	AUPR
1	Drug Average	0.884	0.746
	tCNNS	0.878 ± 0.001	0.735 ± 0.004
	GraphDRP	0.8830 ± 0.0009	0.7468 ± 0.0009
	DeepTTA	0.9101 ± 0.0008	0.799 ± 0.002
	BinaryET	**0.9158 ± 0.0006**	**0.8084 ± 0.0002**
2	Drug Average	0.877	0.761
	tCNNS	0.879 ± 0.003	0.766 ± 0.001
	GraphDRP	0.881 ± 0.002	0.774 ± 0.003
	DeepTTA	**0.920 ± 0.002**	**0.841 ± 0.004**
	BinaryET	0.919 ± 0.003	0.840 ± 0.005
3	Drug Average	0.886	0.765
	tCNNS	0.879 ± 0.001	0.757 ± 0.003
	GraphDRP	0.8829 ± 0.0006	0.765 ± 0.002
	DeepTTA	0.9212 ± 0.0008	0.837 ± 0.001
	BinaryET	**0.9239 ± 0.0005**	**0.840 ± 0.001**

Table 21. Ablation study for DeepTTA for multiple split types and testing type, the table shows that removing the drug branch decreases performance. For given split and testing time the **bold** metric gives the best performance between DeepTTA and DeepTTA-DB.

Strat type	Train test split	DeepTTA AUC	DeepTTA-DB AUC	DeepTTA AUPR	DeepTTA-DB AUC
Drug strat	1	**0.747 ± 0.008**	0.739 ± 0.006	**0.53 ± 0.02**	**0.53 ± 0.01**
	2	**0.785 ± 0.002**	0.777 ± 0.009	**0.620 ± 0.008**	0.60 ± 0.02
	3	**0.789 ± 0.005**	0.785 ± 0.007	**0.601 ± 0.003**	0.6 ± 0.008
CL strat	1	**0.926 ± 0.002**	0.924 ± 0.001	**0.814 ± 0.003**	0.812 ± 0.002
	2	**0.934 ± 0.002**	0.931 ± 0.003	**0.845 ± 0.003**	0.840 ± 0.005
	3	**0.932 ± 0.001**	0.9308 ± 0.0007	**0.842 ± 0.002**	0.8390 ± 0.0005
No strat	1	**0.9101 ± 0.0008**	0.909 ± 0.002	**0.799 ± 0.002**	**0.799 ± 0.003**
	2	**0.920 ± 0.002**	0.917 ± 0.003	**0.841 ± 0.004**	0.836 ± 0.005
	3	**0.9212 ± 0.0008**	0.9196 ± 0.0009	**0.837 ± 0.001**	0.834 ± 0.001

Appendix K. Binary mixed set testing

This section shows the tables for mixed set testing for binary response values.

Table 22. Mixed set testing for train test split 2 and 3 with binary response values.

Methods	AUC	AUPR
Marker	0.9286 ± 0.0003	0.8691 ± 0.0005
tCNNS	0.9263 ± 0.0009	0.864 ± 0.002
GraphDRP	0.9354 ± 0.0004	0.880 ± 0.002
DeepTTA	0.9406 ± 0.0009	0.891 ± 0.003
BinaryET-DB	0.9451 ± 0.0005	0.8975 ± 0.0009
BinaryET	**0.946 ± 0.001**	**0.9 ± 0.002**
Marker	0.9284 ± 0.0001	0.8648 ± 0.0003
tCNNS	0.9244 ± 0.0006	0.8576 ± 0.0007
GraphDRP	0.9339 ± 0.0005	0.875 ± 0.001
DeepTTA	0.9435 ± 0.0002	0.8923 ± 0.0002
BinaryET-DB	0.9455 ± 0.0007	0.896 ± 0.001
BinaryET	**0.9466 ± 0.0007**	**0.898 ± 0.001**

References

S. Kim, J. Chen, T. Cheng, A. Gindulyte, J. He, S. He, Q. Li, B. A. Shoemaker, P. A. Thiessen, B. Yu, et al. Pubchem 2023 update. Nucleic Acids Research, 51(D1):D1373–D1380, 2023.

R. A. Irizarry, B. Hobbs, F. Collin, Y. D. Beazer-Barclay, K. J. Antonellis, U. Scherf, and T. P. Speed. Exploration, normalization, and summaries of high density oligonucleotide array probe level data. Biostatistics, 4(2):249–264, 2003
